# The Adjuvant Effects of High-Molecule-Weight Polysaccharides Purified from *Antrodia cinnamomea* on Dendritic Cell Function and DNA Vaccines

**DOI:** 10.1371/journal.pone.0116191

**Published:** 2015-02-27

**Authors:** Chi-Chen Lin, I-Hong Pan, Yi-Rong Li, Yi-Gen Pan, Ming-Kuem Lin, Yi-Huang Lu, Hsin-Chieh Wu, Ching-Liang Chu

**Affiliations:** 1 Institute of Biomedical Sciences, National Chung Hsing University, Taichung, Taiwan; 2 Rong Hsing Research Center for Translational Medicine, National Chung Hsing University, Taichung, Taiwan; 3 Department of Medical Research and Education, Taichung Veterans General Hospital, Taichung, Taiwan; 4 Biomedical Technology and Device Research Laboratories, Industrial Technology Research Institute, Hsinchu, Taiwan; 5 Graduate Institute of Immunology, College of Medicine, National Taiwan University, Taipei, Taiwan; 6 Department of Chinese Pharmaceutical Sciences and Chinese Medicine Resources, China Medical University, Taichung, Taiwan; Istituto Superiore di Sanità, ITALY

## Abstract

The biological activity of the edible basidiomycete *Antrodia cinnamomea* (AC) has been studied extensively. Many effects, such as anti-cancer, anti-inflammatory, and antioxidant activities, have been reported from either crude extracts or compounds isolated from AC. However, research addressing the function of AC in enhancing immunity is rare. The aim of the present study is to investigate the active components and the mechanism involved in the immunostimulatory effect of AC. We found that polysaccharides (PS) in the water extract of AC played a major role in dendritic cell (DC) activation, which is a critical leukocyte in initiating immune responses. We further size purified and identified that the high-molecular weight PS fraction (greater than 100 kDa) exhibited the activating effect. The AC high-molecular weight PSs (AC hmwPSs) promoted pro-inflammatory cytokine production by DCs and the maturation of DCs. In addition, DC-induced antigen-specific T cell activation and Th1 differentiation were increased by AC hmwPSs. In studying the molecular mechanism, we confirmed the activation of the MAPK and NF-κB pathways in DCs after AC hmwPSs treatment. Furthermore, we demonstrated that TLR2 and TLR4 are required for the stimulatory activity of AC hmwPSs on DCs. In a mouse tumor model, we demonstrated that AC hmwPSs enhanced the anti-tumor efficacy of the HER-2/neu DNA vaccine by facilitating specific Th1 responses. Thus, we conclude that hmwPSs are the major components of AC that stimulate DCs via the TLR2/TLR4 and NF-κB/MAPK signaling pathways. The AC hmwPSs have potential to be applied as adjuvants.

## Introduction

Edible fungi have nutritional and medicinal values and can be the functional food in dietary supplements [1]. Many edible fungi belong to the Aphyllophorales (polypore), a large group of terrestrial fungi of the phylum Basidiomycota (basidiomycetes) [2]. Recently, a precious basidiomycete, *Antrodia cinnamomea* [syn. *A*. *camphorata*] (AC), became a popular health food for reducing cancer and improving liver function in Taiwan [3]. The effects of the bioactive components extracted or purified from AC have been studied extensively; however, the majority of investigations focus on their anti-cancer and anti-inflammatory effects [4]. The immunostimulatory effect of AC remains to be explored.

Polysaccharides (PSs) are well-known bioactive compounds and broadly exist in living organisms. A great deal of PSs has been isolated from the fruiting bodies, cultured mycelia and the culture filtrates of basidiomycetes [5]. Many functions of dietary PSs have been identified, including anti-cancer, anti-inflammatory, antioxidant, and immunomodulatory effects [6]. These biological activities of PSs are closely correlated to their physicochemical properties, such as molecular size, types and ratios of constituent monosaccharides, and features of glycosidic linkages [7]. Recently, studying the mechanism of the immunomodulatory effect of PSs has become a major topic in glycobiology and immunology. The PSs of AC have been shown to contain galactose, glucose, fructose, xylose and fucose [8]. In the high molecular weight fraction (> 100 kDa), the AC PSs have a backbone built of partial fucosylated 1,6-α-D-mannogalactan composed of a nonadecasaccharide repeating unit with myo-inositol, sorbitol, fucose, galactosamine, glucosamine, galactose, glucose, mannose, and/or fructose substitutions [9]. However, the immunomodulatory activity of AC PSs is largely unknown.

The immune system plays the most essential role in maintaining human health against infectious agents and tumor cells. There are many types of immune cells in this system. A specialized leukocyte known as the dendritic cell (DC) is critical in initiating immune responses [10]. They are in their immature state in peripheral tissues and become mature when pathogens are recognized by the pattern recognition receptors (PRRs) on DCs [11]. Mature DCs then migrate to secondary lymphoid organs where they present antigens to naive T cells. The activated DCs up-regulate the expression of major histocompatibility complex (MHC) and co-stimulatory molecules for T cell activation and secrete a variety of cytokines and chemokines for T cell differentiation and recruitment [12]. Due to their key role in the immune system, DCs have being applied as potent vaccines in treating cancer and viral infections [13,14]. Thus, natural or synthetic substances that can stimulate DCs may potentially be developed into adjuvants and applied to immunotherapy and vaccination.

In this study, we examined the immunostimulatory effect of AC on mouse bone marrow-derived DCs (BMDCs). Our results showed that the PSs of AC possessed the ability to promote DC activation and function. Subsequently, we determined that the activating effect on DCs was mainly achieved by the high molecular weight PSs (hmwPSs) of AC. Furthermore, the AC hmwPSs enhanced the therapeutic effect of a DNA vaccine in a mouse tumor model. Thus, we concluded that the AC hmwPSs may be an immunopotentiator and candidate for use as a vaccine adjuvant.

## Materials and Methods

### Preparation and subfractions of AC PSs

The fruiting bodies of *A*. *cinnamomea* (10 g) were treated with distilled water (100 mL) at 100°C for 1 h. The water extract was centrifuged, filtered through the Toyo No. 1 filter paper (Toyo roshi kaisha, Ltd., Tokyo, Japan), and concentrated with a rotary evaporator (R-210, Buchi Labortechnik AG, Flawil, Switzerland). After centrifugation, the supernatant was dialyzed extensively against deionized water, mixed with ethanol (final 30%), and then chilled at 4°C for 6 h to precipitate contaminated nucleic acids and proteins. Next, the extract was mixed with ethanol (80%, v/v), incubated overnight at 4°C, and centrifuged to obtain the crude PSs. For further fractionation, the crude PSs were partially separated into 6 fractions by Amicon Ultra-15 5K, 10K, 30K, 50K, 100K centrifugal filter devices (Millipore, County Cork, Ireland). Briefly, crude PSs (2 g) were suspended with distilled water (20 mL) and centrifuged. The supernatant (10 mL) was applied to a 100K filter unit of a centrifugal filter device. After centrifugation, the 100K filter was transferred to a new tube and PSs (> 100 kDa) were eluted by centrifugation with the filter upside down, and the PSs were then lyophilized. Repeating the same protocol, we obtained 5 other fractions of PSs by serially decreasing the filter sizes (50K, 30K, 10K, and 5K). The percentages and weights of each fraction are shown in Table 1. To remove nucleic acids and proteins, nuclease treatment and 30% ethanol precipitation were repeated until no significant amounts of nucleic acid and protein determined by UV absorption (OD at 260 nm for nucleic acid and OD at 280 nm for protein). To remove endotoxin, the samples were passed through an EndoTrap Blue column (Hyglos, Bernried, Germany) and then analyzed by the limulus amebocyte lysate test (Cambrex Bio Science Walkersville, Inc., Walkersville, MD, USA). The endotoxin level was less than 1 ng (10 EU)/mg extract.

**Table 1:** The weight and percentage of each fraction in AC PSs after purification.

MW (Da) of fraction	Water insoluble	>100K	100K~50K	50K~30K	30K~10K	10K~5K	<5K
**Weight (mg)**	96.3	915.9	308.0	212.9	155.7	47.3	229.9
**%**	4.90	46.59	15.67	10.83	7.92	2.40	11.69

To further exclude any contamination effect in AC PS samples, we pre-treated the samples with polymyxin B (10 μg/mL) at 37°C for 2 h to inhibit the endotoxin activity. In another experiment, we pre-treated AC PSs (100 mg) with 5 mL of 2M trifluoroacetic acid (TFA) and hydrolyzed at 95°C for 16 h to destroy PSs [15]. These materials were used in supporting information.

### Mice

C57BL/6, C3H/HeN, and TLR4 mutant (C3H/HeJ) mice were purchased from National Laboratory Animal Center (NLAC, Taipei, Taiwan) and National Cheng-Kung University (NCKU, Tainan, Taiwan). OT-I and OT-II TCR transgenic mice were provided by Dr. Clifford Lowell (UCSF, San Francisco, CA). TLR2-deficient mice were provided by Dr. Chih-Peng Chang (NCKU, Tainan, Taiwan). All of the animals were kept in a specific pathogen-free facility at Taichung Veterans General Hospital (Taichung, Taiwan) and handled according to protocols approved by the Institutional Animal Care and Use Committee.

### Ethics statement

All animal experiments were performed according to the protocols approved by the Animal Care and Use Committee of Taichung Veterans General Hospital (Taichung, Taiwan). Mice were euthanized with CO_2_ inhalation in approved chambers. We made all efforts to minimize animal suffering and discomfort in accordance with the recommendations in the Guide for the Care and Use of Laboratory Animals of the National Institutes of Health, USA.

### Preparation of mouse DCs

Mouse DCs were derived from the bone marrow of mice as previously described [16]. Briefly, bone marrow cells were isolated from the femurs and tibia of mice. After removing the red blood cells, the cells were then seeded in 24-well plates with complete RPMI 1640 medium (L-glutamine, nonessential amino acids, sodium pyruvate, HEPES, 2-ME, penicillin/streptomycin) supplemented with 10% FBS and 10 ng/mL recombinant mouse GM-CSF (PeproTech). On day 7, the DCs (> 70%) were collected for analysis.

### Preparation of human monocyte-derived DCs (MoDCs)

Human DCs were generated from peripheral blood mononuclear cells (PBMCs) as described previously [17]. Briefly, PBMCs were sorted from healthy volunteer donors by magnetic anti-CD14 MicroBeads according to the manufacturer’s protocol (Miltenyi Biotec). Then, the purified monocytes (> 90% CD14^+^ cells) were cultured in 6-well plates with complete RPMI 1640 medium (Gibco) containing 10% FBS (Gibco), recombinant human (rh)GM-CSF (80 ng/mL, PeproTech), and rhIL-4 (100 ng/mL, PeproTech) to generate DCs. Fresh media containing GM-CSF and IL-4 were added every two days. The non-adherent cells were collected after 7 days and washed with PBS before using in the experiments.

### Detection of cytokine production

DCs were treated with AC PSs (crude extract or various fractions), LPS (100 ng/mL, from *Escherichia coli* serotype O26:B6, Sigma, St. Louis, MO, USA), or lipoteichoic acid (LTA, 1 μg/mL, from *Staphylococcus aureus*, Sigma) for 24 h. The production of cytokines (TNF-α, IL-6, and IL-12) was quantified by ELISA (eBioscience and R&D systems) as described previously [18].

### Measurement of DC maturation

DCs were treated with PBS (control) or AC hmwPSs (5 or 10 μg/mL) for 16 h. Maturation was determined by measuring the expression of MHC and co-stimulatory molecules as described previously [19]. After treatment, cells were collected and immunostained with fluorescent dye-conjugated mAbs specific for mouse CD11c, I-A^b^, H-2^b^, CD40, CD80, CD86 (Biolegend), or for human CD1a, HLA-DR, CD40, CD83 (Biolegend), and then analyzed by flow cytometry. The mean fluorescence intensity (MFI) is indicated in each graph.

### Assay for OVA-specific T cell activation

The DC-induced OVA-specific T cell activation was determined as described previously [20]. Briefly, DCs were incubated with OVA_257–264_ (for OT-I) or OVA_323–339_ (For OT-II) (1 μg/mL, synthesized by Echo Chemical Co., Taiwan) in the presence or absence of AC hmwPSs (10 μg/mL) for 3 h, and then OT-I or OT-II T cells were added at a 1:5 ratio of DC:T cells to the culture. T cell proliferation was measured by ^3^H-thymidine incorporation after 72 h. To detect IFN-γ production, supernatants were collected from the DC/T cell cultures and the IFN-γ production was measured by ELISA (eBioscience).

### Detection of MAPK activation

The MAPK activity was determined by Western blot. Briefly, DCs were treated with AC hmwPSs (10 μg/mL), and the total protein was extracted using lysis buffer (50 mM Tris/HCl; 150 mM NaCl; 1% (v/v) Nonidet P-40; 0.25% (w/v) sodiumdeoxycholate; 1 μg/mL leupeptin; 1 μg/mL pepstatin; 1 μg/mL aprotinin; 1 mM PMSF; 1 mM EDTA; 1 mM NaF, and 1 mM Na3 VO4). The protein concentration was measured using a BCA protein assay kit (Pierce, Rockford, IL, USA). Then, proteins were mixed with sample buffer, boiled, and separated on 10% SDS polyacrylamide gel. All of the proteins were transferred to nitrocellulose membrane and blocked in TBS with 0.1% (v/v) Tween 20 and 5% (w/v) non-fat dry milk. After blocking, the membranes were incubated with primary antibodies against ERK1/2 (no. 9102), p38 (no. 9212), JNK (SC- 571, Santa Cruz Biotechnology, Danvers, MA, USA), β-Actin (no. 4970), and their phosphorylated forms using phospho-ERK1/2 (no. 9101), phospho-p38 (no. 9211), phospho-JNK (no. 4688) (all purchased from Cell Signaling Technology). Subsequently, the blots were washed and incubated with secondary HRP-conjugated goat anti-mouse or goat anti-rabbit antibody (Jackson ImmunoResearch, PA, USA). Finally, the blots were washed and developed by enhanced chemiluminescence (GE Healthcare, UK) and analyzed using the LAS3000 system (Fujifilm, Tokyo, Japan). Densitometric analysis was performed with ImageJ software (National Institute of Health, Bethesda, MD, USA).

### Analysis of NF-κB activity

For detection of NF-κB activity, we prepared the nuclear extracts first. DCs were treated with PBS (control), LPS (100 ng/mL), or AC hmwPSs (10 μg/mL), and the nuclear extracts were prepared using the NE-PER Nuclear and Cytoplasmic Extraction system (Pierce Rockford, IL, USA), according to manufacturer’s instruction. Protein concentrations were determined using a BCA protein assay kit. For each assay, 5 mg/mL of nuclear extracts was used in a TransAM NF-κB p65 ELISA kit (Active Motif, Carlsbad, CA), according to the manufacturer’s instruction.

### Analysis of DC maturation *in vivo*

As described previously [21], we collected cells from DNase I (fraction IX; Sigma)- and collagenase D (Roche Diagnostics, Mannheim, Germany)-digested inguinal lymph nodes and spleens of mice (n = 5) after intraperitoneal treatment of PBS or AC hmwPSs (10 μg/mouse) for 6 h. Then, CD11c^+^ DCs were purified with CD11c (N418) Microbeads and LS separation columns (Miltenyi Biotec, Auburn, CA, USA) according to the manufacturer’s instructions. The expressions of CD40, CD86 and MHC class II were analyzed by flow cytometry. To determine the IL-12 production, quantitative real-time PCR was performed with the primer pair for IL-12 as shown in previous report. The expression of IL-12 mRNA of DCs from AC hmwPSs-treated mice was presented as relative fold to PBS-treated group.

### The HER-2/neu DNA vaccine on a mouse model with p185neu-expressing MBT-2 tumor

The mouse tumor model was established by implanting with a p185neu-expressing MBT-2 tumor and immunizing with the HER-2/neu DNA vaccine, as previously described [20]. Briefly, p185neu-expressing MBT-2 cells were injected subcutaneously into the flank of C3H/HeN mice. After ten days, the mice were immunized with naked HER-2/neu DNA vaccine using a low pressure-accelerated gene gun (Bioware, Technologies Co. Ltd, Taipei, Taiwan) three times in one week. In co-administration with AC hmwPSs, the PSs were intraperitoneally injected at the immunization site after vaccination. The anti-tumor effect of vaccination was then determined by monitoring the size of the p185neu-expressing MBT-2 tumor every three days. Tumor size was calculated using the formula for a rational ellipse: (m_1_ × m_2_ × m_2_ × 0.5236), where m_1_ represents the longer axis and m_2_ the shorter axis. The mice were sacrificed when the tumor size was greater than 2,500 mm^3^ in volume.

### Analysis for Th1 responses in mice immunized with HER-2/neu DNA vaccine

The Th1 responses were determined by intracellular staining for IFN-γ production by CD8^+^ T cells. The inguinal lymph node cells were isolated from mice with various treatments and stimulated with recombinant HER-2/neu protein (10 μg/mL, R&D System) in complete RPMI 1640 medium overnight. The cells were stained with anti-CD8 and anti-IFN-γ antibodies and then analyzed by flow cytometry. The percentage of CD8^+^IFN-γ^+^ cells in total CD8^+^ T cells is shown in each graph. In addition, the amount of IFN-γ and IL-4 was measured by qPCR. CD4^+^ T cells were purified using the Dynal CD4 Negative Isolation Kit (Dynal AS, Oslo, Norway). Then, total RNA was prepared using the TRIzol reagent (Invitrogen, Carlsbad, CA, USA), and cDNA was generated using oligo dT as primer and reverse transcriptase (Promega, Madison, Wisconsin, USA) according to the manufacturer’s instructions. Primer pairs for IFN-γ, IL-4, and hypoxanthine guanine phosphoribosyl transferase 1 (HPRT) were used as described previously [20]. ABI 7500 Fast Real-Time system with SYBR Green PCR Master Mix (Applied Biosystems) was used for quantitative real-time PCR. The expressions of IFN-γ and IL-4 were normalized with HPRT expression. The data are shown as fold change compared to the vector only control group.

### Data analysis

Significance of the AC PS treatment in comparison with control PBS treatment or HER-2/neu DNA vaccine immunization in comparison with vector alone vaccination was calculated using Mann–Whitney U test. Survival was analyzed by Kaplan–Meier analysis. We used the GraphPad Prism software (v.5.00, GraphPad Software Inc., San Diego, CA). P-values less than 0.05 were considered significant.

## Results

### Identification of high molecular weight PSs (hmwPSs) as major fraction of AC for DC activation

**Fig 1:**
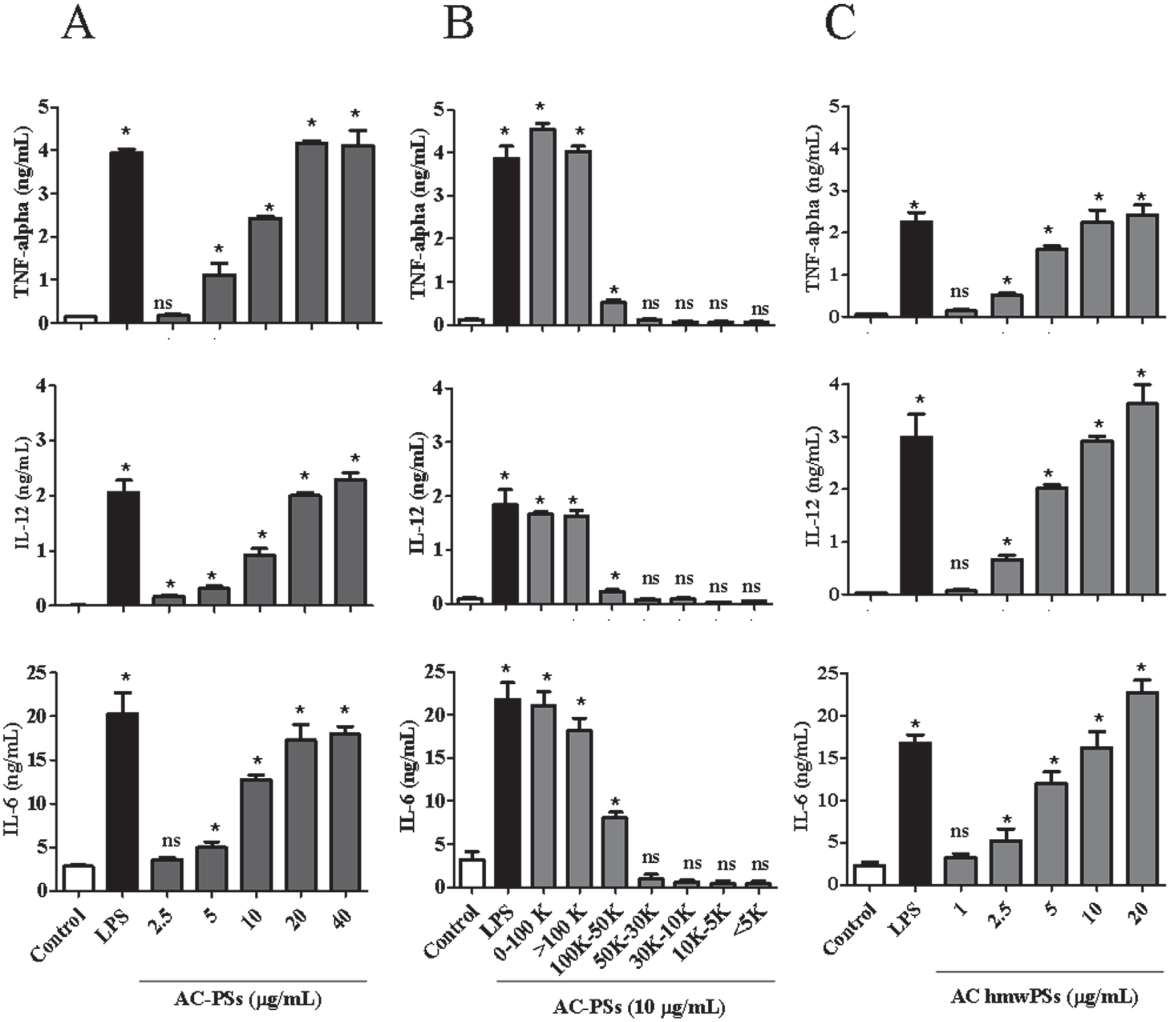
The hmwPSs were the major fraction of AC for DC activation. DCs were treated with PBS (control), LPS (100 ng/mL), or various doses of AC PSs as indicated. Supernatants were collected after 24 h (6 h for TNF-α). The amounts of TNF-α, IL-6, and IL-12 were determined by ELISA. (A) The effect of crude AC PSs in the first water extraction. (B) The effect of each fraction of AC PSs with different molecular weights. (C) The effect of hmwPS fraction. The data shown are the mean + SD of three samples. ^NS^ *p*>0.05; *p<0.05 (*Mann*–*Whitney U test*) are comparisons between LPS- or AC PS-treated and PBS-treated DCs. All of the results are representative of three or four independent experiments.

Various activities of the PSs isolated from AC have been reported; however, the enhancement of immune responses has not been examined. Therefore, we prepared a crude extract of AC PS for testing its effect on DCs, which play a critical role in initiating immune responses. The crude AC PSs promoted the dose-dependent production of TNF-α, IL-6, and IL-12 by DCs, which is a hallmark of DC activation (Fig. 1A). These results suggest that AC PSs may have stimulatory effects on DCs.

To verify the major PS components responding to DC activation, we further purified the PSs from crude extract by molecular weight, as shown in Table 1. Six fractions were obtained and their effects on DC activation were analyzed. As expected, the fraction containing the hmwPSs (> 100 kDa) had the strongest induction of TNF-α, IL-6, and IL-12 production by DCs compared to other low molecular weight PS (lmwPS, < 100 kDa) fractions (Fig. 1B). Thus, we determined that hmwPSs were very likely the major components in the total PSs of AC for DC activation.

To confirm that the stimulatory effect of crude AC PSs on DCs was mainly contributed by AC hmwPSs, we determined the ability of AC hmwPSs to induce cytokine production by DCs, as shown in Fig. 1A. We found that AC hmwPSs also augmented the secretion of TNF-α, IL-6, and IL-12 by DCs in a dose-dependent manner (Fig. 1C), and the saturated dose (approximately 10 μg/mL) was about half compared to the dose of crude AC PSs (approximately 20–40 μg/mL, Fig. 1A). This was consistent with the percentage of AC hmwPSs in the total AC PSs (approximately 47%, Table 1). Simultaneously, we excluded the possible endotoxin contamination because polymyxin B did not significantly reduce the activity of AC hmwPSs compared to the inactivation of LPS (S1 Fig.). In addition, the TFA-destroyed hmwPSs lost the stimulatory activity, suggesting their ability to activate DCs is intrinsic (S2 Fig.). These data clearly indicated that the AC hmwPSs can activate DCs and may be potent immunostimulators.

### AC hmwPSs promoted DC maturation

**Fig 2:**
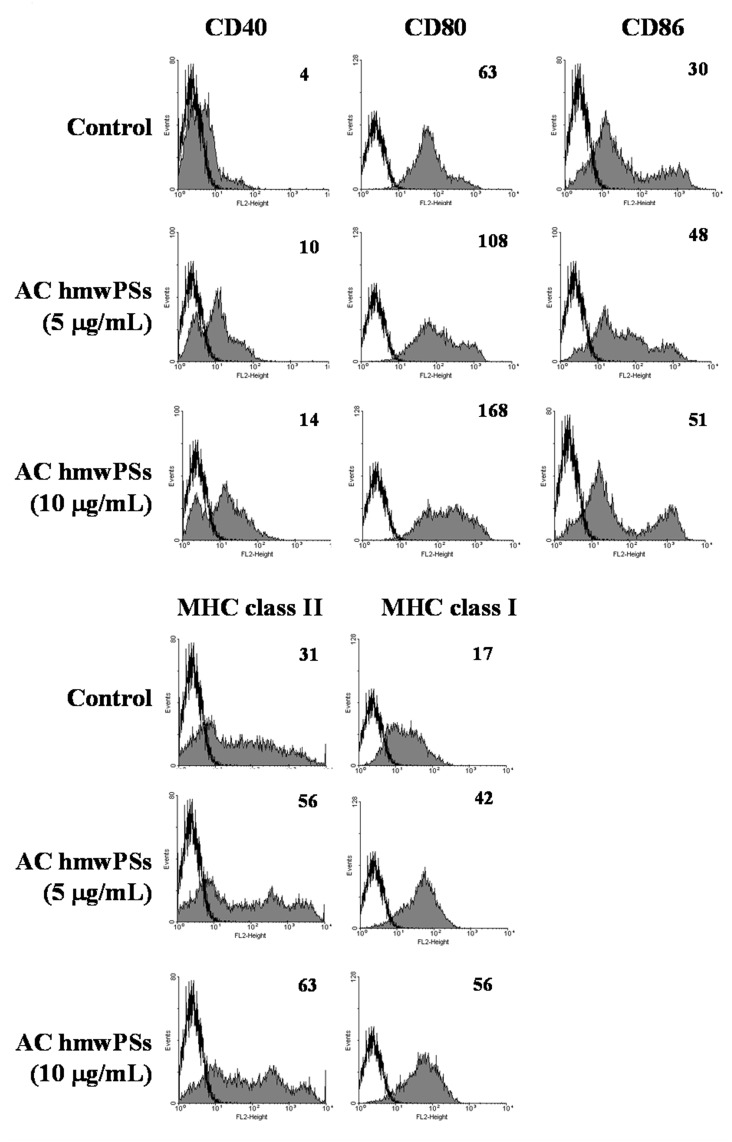
AC hmwPSs promoted DC maturation. DCs were treated with PBS (control) or AC hmwPSs (5 or 10 μg/mL) for 16 h. The expressions of MHC class I & II, CD40, CD80, and CD86 (gray-filled area) were determined by immunostaining and flow cytometry. All of the data shown were gated on CD11c^+^ cells. The black line represents staining with an isotype-matched control antibody. The level of expression is indicated as mean fluorescence intensity (MFI) in each graph. All of the results are representative of three or four independent experiments.

Maturation is a critical process for DC function to initiate adaptive immunity. Therefore, we evaluated the effect of AC hmwPSs on DC maturation. Upon AC hmwPS treatment, the expression levels of co-stimulatory molecules (CD40, CD80, and CD86) and MHCs (class I and II) were increased in DCs, indicating that the DCs became mature (Fig. 2). We also checked the effects of AC lmwPSs on DC maturation but no enhancement of CD40 and CD86 expression was observed (S3 Fig.). These results showed that the AC hmwPSs promote DC maturation and then increase the potential of antigen presentation.

### AC hmwPSs also activated human monocyte-derived DCs (MoDCs)

**Fig 3:**
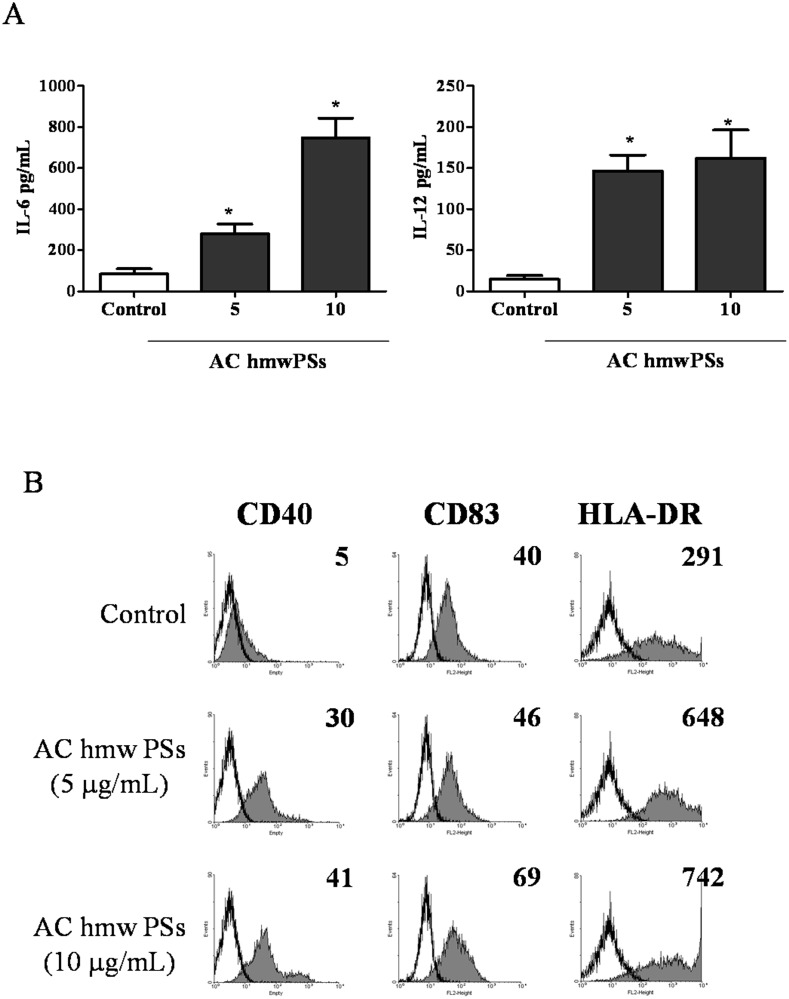
AC hmwPSs activated human MoDCs. Human MoDCs were generated from PBMCs and treated with PBS (control) or AC hmwPSs (5 or 10 μg/mL). (A) After 24 h, supernatants were collected. The amounts of IL-6 and IL-12 were measured by ELISA. Data are presented as the mean + SD of three samples. *p<0.05 (*Mann*–*Whitney U test*) are comparisons between AC hmwPS-treated and PBS-treated MoDCs. (B) After 16 h, the expressions of CD40, CD83 and HLA-DR (gray-filled area) were determined by immunostaining and flow cytometry. All data shown were gated on CD1a^+^ cells. The black line represents staining with an isotype-matched control antibody. The level of expression is indicated as MFI in each graph. All data are representative of five independent experiments with cells from different donors.

To evaluate the availability of AC hmwPSs in clinical application, we examined the stimulatory effect of AC hmwPSs on human MoDCs. MoDCs were generated from PBMCs and then treated with PBS or AC hmwPSs for 24 h. AC hmwPSs significantly induced IL-6 and IL-12 production by MoDCs (Fig. 3A). In addition, AC hmwPSs up-regulated the expression levels of CD40, CD83 and HLA-DR in MoDCs compared to PBS control (Fig. 3B). We still observed that AC lmwPSs did not stimulate MoDCs (data not shown). Thus, we confirmed that the AC hmwPSs also promote human DC activation and could possibly be applied in clinical in the future.

### AC hmwPSs facilitated DC-induced T cell activation and Th1 differentiation

**Fig 4:**
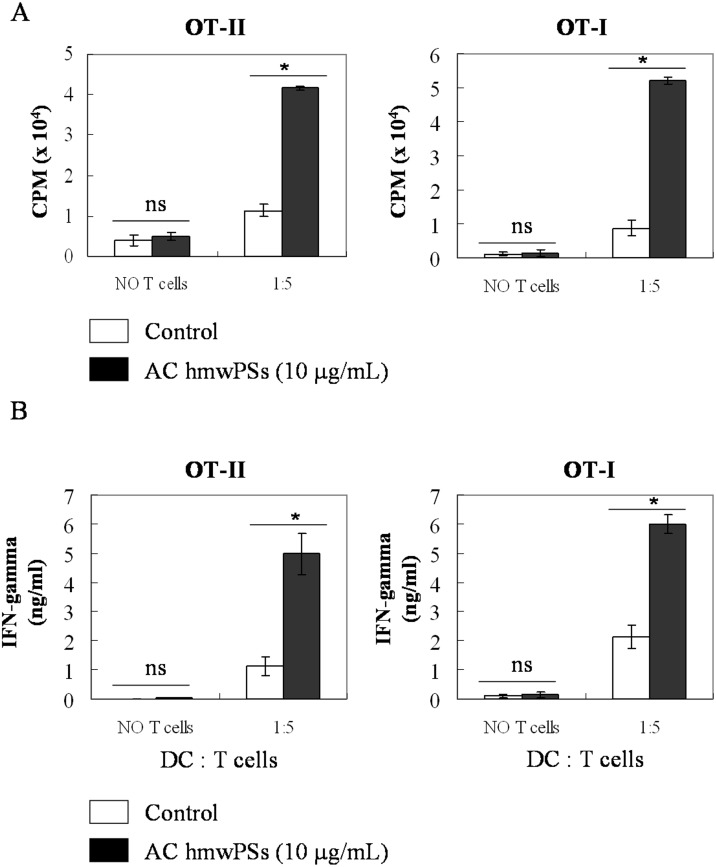
AC hmwPSs facilitated DC-induced T cell activation and Th1 differentiation. OT-I or-II T cells were co-cultured with PBS- (white bar) or AC hmwPS-treated (10 μg/mL, black bar) DCs pulsed with OVA_257–264_ (for OT-I) or OVA_323–339_ (For OT-II) peptides at the indicated ratio of DC:T cell. (A) T cell proliferation was determined by ^3^H-thymidine incorporation after 3 days. (B) The supernatants were collected from the cultures after 4 days. IFN-γ production was measured by ELISA. The data are the mean + SD of three samples. ^NS^ *p*>0.05 (*Mann*–*Whitney U test*) are comparisons between AC hmwPS-treated and PBS-treated DCs. All of the results are representative of three independent experiments.

Activation of naive T cells is initiated by mature DCs, so we examined whether AC hmwPSs affect the ability of DCs to induce T cell activation. DCs were treated with PBS or AC hmwPSs and loaded with OVA_257–264_ or OVA_323–339_ peptide, and then co-cultured with OT-I or OT-II T cells. As shown in Fig. 4A, AC hmwPSs-stimulated DCs increased T cell proliferation compared to control DCs. In addition, we also measured the IFN-γ production by these activated T cells, a hallmark for Th1 differentiation. Significantly, AC hmwPSs-treated DCs induced increased IFN-γ secretion by T cells compared to control DCs (Fig. 4B). Again, the AC lmwPSs had no effect on DCs to activate T cells (data not shown). Our data illustrated that AC hmwPSs augment the ability of DCs to activate Ag-specific T cells and Th1 polarization. This conclusion is in agreement with the enhancement of DC activation and maturation after AC hmwPSs treatment (Figs. 1 and 2).

### Activation of MAPKs and NF-κB by AC hmwPSs in DCs

**Fig 5:**
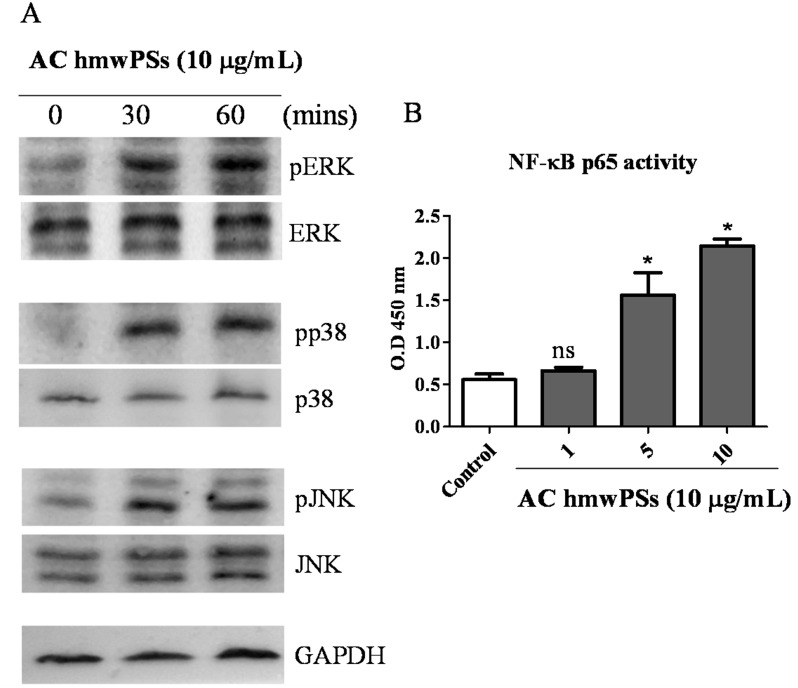
Activation of MAPKs and NF-κB by AC hmwPSs in DCs. DCs were collected and treated with AC hmwPSs (10 μg/mL). Lysis buffer was added at the indicated time points to obtain whole cell lysates or nuclear extracts. (A) The activation of MAPKs was assayed by western blot. Whole cell lysates were incubated with antibodies against phosphorylated protein of ERK, JNK, and p38 MAPK, and then detected with specific 2^nd^ antibodies and ECL system. The loading controls are shown and represent the use of antibodies against the total protein of these MAPKs. The data are representative of three independent experiments. (B) The activation of NF-κB was determined by p65 binding assay. The NF-κB binding activity in nuclear extract was measured using TransAM NF-κB p65 ELISA kit and shown as value read at OD450. The data are the mean + SD of three samples. ^NS^ *p*>0.05 (*Mann*–*Whitney U test*) are comparisons between LPS- or AC hmwPS-treated and PBS-treated DCs. All of the results are representative of three independent experiments.

Next, we explored the molecular mechanism involved in the DC activation by AC hmwPSs. MAPK and NF-κB pathways have been identified to be critical in mediating activation signals in DCs. Therefore, we checked the status of MAPK and NF-κB in DCs after treatment with AC hmwPSs. As shown in Fig. 5, AC hmwPSs induced the phosphorylation of MAPKs (ERK, p38, and JNK) (Fig. 5A) and the activation of NF-κB p65 (Fig. 5B), which indicated that both pathways were activated by AC hmwPSs resulting in DC activation and maturation. As we expected, AC lmwPSs could not activate these signaling molecules (data not shown).

### TLR2 and TLR4 were required in DC activation by AC hmwPSs

**Fig 6:**
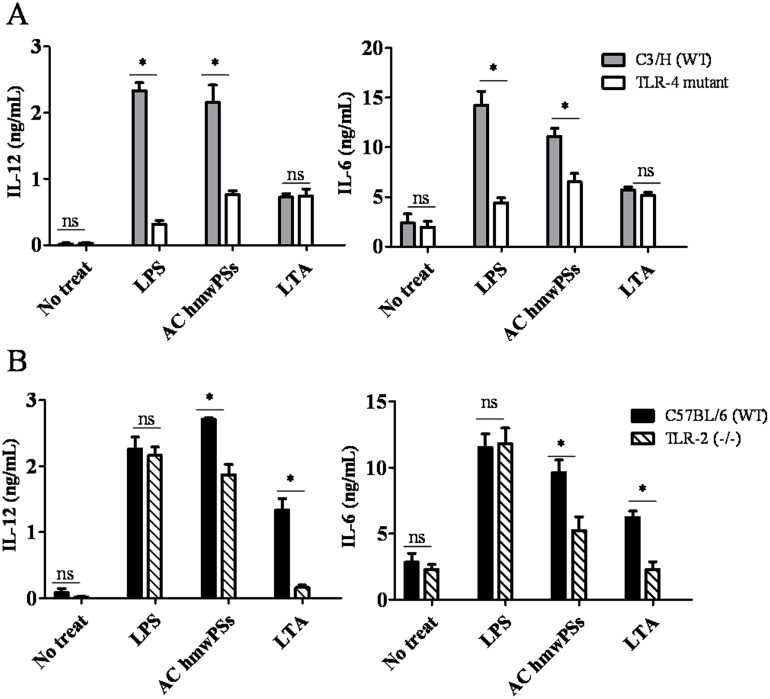
TLR2 and TLR4 were involved in DC activation by AC hmwPSs. DCs were generated from (A) C3H/HeN and C3H/HeJ (TLR4 mutant), or (B) C57BL/6 and TLR-2 KO mice, and then treated with PBS (control), LPS (100 ng/mL), LTA (1 μg/mL), or AC hmwPSs (10 μg/mL). The supernatants were collected after 24 h. The levels of IL-6 and IL-12 were determined by ELISA. The data shown are the mean + SD of three samples. ^NS^ *p*>0.05; *p<0.05 (*Mann*–*Whitney U test*) are comparisons between LPS-, LTA- or AC hmwPS-treated and PBS-treated DCs. All of the results are representative of three independent experiments.

The MAPK and NF-κB pathways can be triggered by many receptors on the DC surface. TLRs are the most important receptor to sense microbes in environment. TLR2 and TLR4 have been revealed to participate in the DC activation by several sources of PSs. Thus, we asked whether these two receptors also participate in the stimulation of AC hmwPSs on DCs. We generated BMDCs from TLR2^-/-^ and TLR4 mutant mice and then stimulated with AC hmwPSs. Compared to DCs derived from wild type (WT) mice, TLR4 mutant DCs secreted less IL-6 and IL-12 after AC hmwPS treatment (Fig. 6A). The reduction of cytokines in TLR2^-/-^ DCs was not as strong as that in TLR4 mutant DCs; however, it was still significant (Fig. 6B). We could not detect the cytokine production by AC lmwPSs-treated DCs from WT and mutant mice (data not shown). According to the results, we concluded that AC hmwPSs activate DCs via both TLR2 and TLR4 recognition.

### AC hmwPS increased DC activation *in vivo*

**Fig 7:**
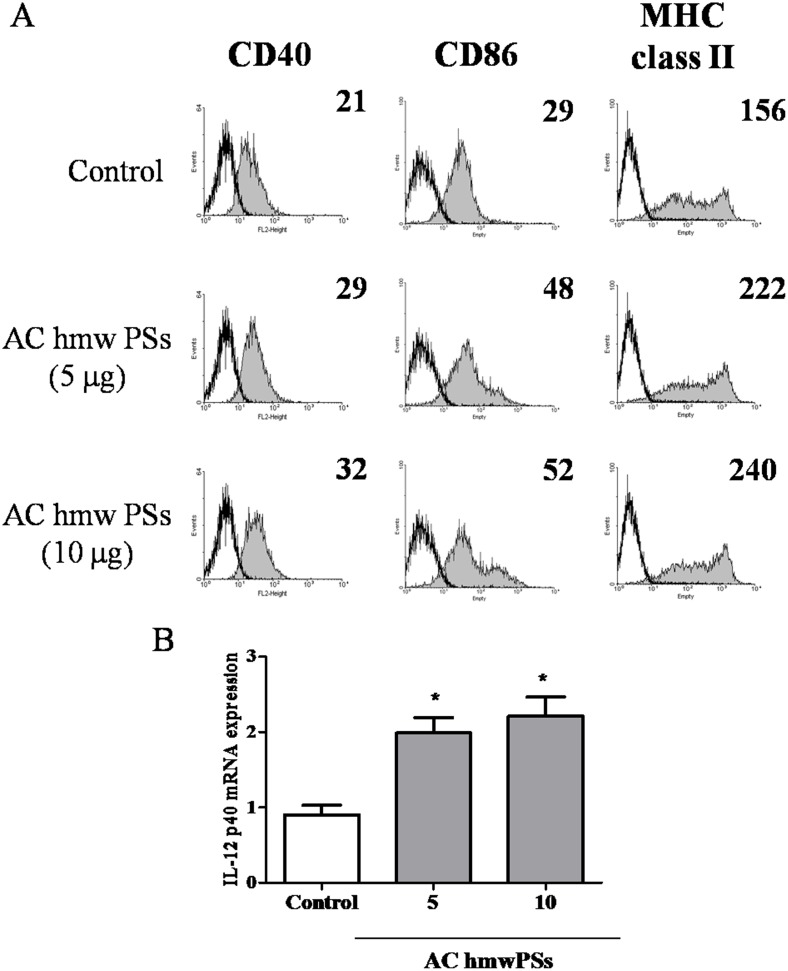
AC hmwPSs promoted DC activation *in vivo*. CD11c^+^ DCs were isolated from draining lymph nodes and spleen of mice intraperitoneally injected with PBS (control) or AC hmwPSs (5 or 10 μg/mouse) after 6 h. (A) The expressions of CD40, CD86 and MHC class II (gray-filled area) were determined by immunostaining and flow cytometry. The black line represents staining with an isotype-matched control antibody. The level of expression is indicated as MFI in each graph. (B) The amount of IL-12 was determined by quantitative real-time PCR. *p<0.05 (*Mann*–*Whitney U test*) are comparisons between AC hmwPS-treated and PBS-treated mice. All of the results are representative of three or four independent experiments.

We had provided strong evidences for the stimulatory activity of AC hmwPSs on DCs *in vitro*. Therefore, we examined whether AC hmwPSs also promote DC activation *in vivo*. PBS or AC hmwPSs were intraperitoneally injected into mice and CD11c^+^ DCs were collected from draining lymph nodes and spleen after 6 h. As shown in Fig. 7, AC hmwPS treatment resulted in higher expression of CD40, CD86 and MHC class II on DCs (Fig. 7A) and more IL-12 production by DCs (Fig. 7B). The results pointed out the potential application of AC hmwPSs *in vivo*.

### AC hmwPSs strengthened the therapeutic effect of HER-2/neu DNA vaccine in a mouse tumor model

**Fig 8:**
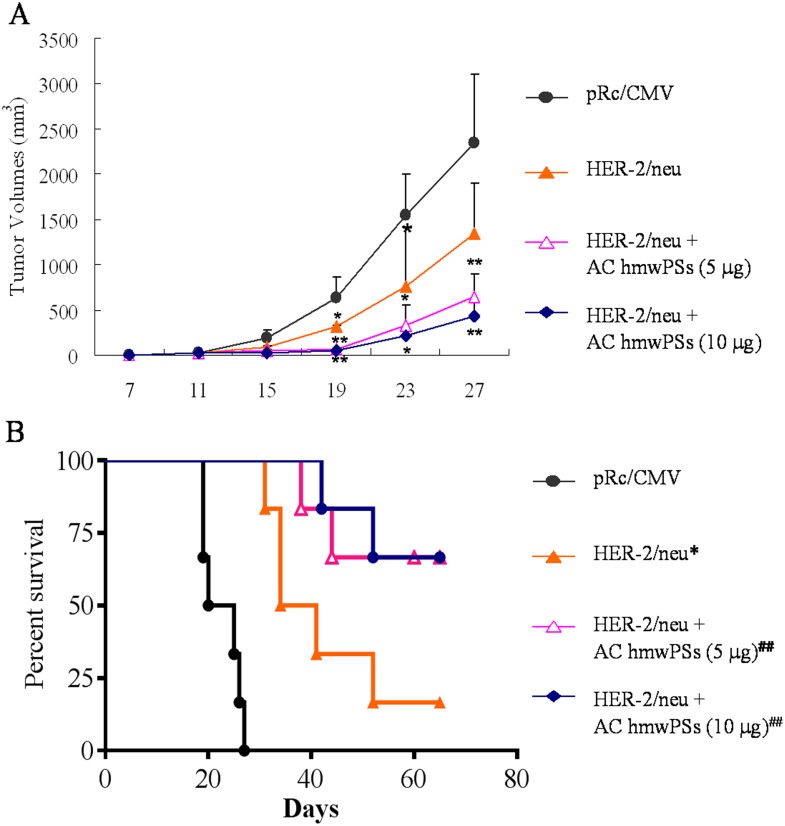
AC hmwPSs enhanced the therapeutic effect of HER-2/neu DNA vaccine in mouse tumor model. The p185neu-expressing MTB-2 cells were inoculated in mice as described in the Materials and Methods. After ten days, the tumor-bearing mice were treated with HER-2/neu DNA vaccine or vaccine combined with AC hmwPSs, as indicated. The data shown are the mean ± SD of six to seven mice per group. (A) Tumor growth curve in mice after vaccination. The tumor volumes were calculated as described in Materials and Methods section at the indicated day. *p<0.05, **p<0.01 (*Mann*–*Whitney U test*) are comparisons of treatment between all vaccinations and vector alone. (B) The Kaplan-Meier survival curve of mice (N = 5) after vaccination. *p<0.05 is *comparisons of treatment between* HER-2/neu DNA *vaccine and vector alone*. ^##^ *p*<0.05 are *comparisons of treatment between* HER-2/neu DNA *vaccine + AC hmwPSs and* HER-2/neu DNA *vaccine*. All of the results are representative of three independent experiments.

We have demonstrated the promoting effect of AC hmwPSs on DC function. In previous studies, we established that the naked HER-2/neu DNA vaccine delivered via gene gun can be used for therapy in p185neu-expressing MBT-2 tumors in C3H/HeN mice [22]. Therefore, we tested the possibility of using AC hmwPSs as adjuvants in this mouse DNA vaccine model. As shown in Fig. 8A, when we immunized the tumor-implanted mice with the control vector alone, the tumors continuously grew in mice. In this case, all mice had to be sacrificed due to the size of their tumors. Treatment of AC hmwPSs alone also had no significant effect in controlling tumor growth (S4 Fig.). In contrast, vaccination with the HER-2/neu DNA alone or HER-2/neu DNA plus AC hmwPSs (5 or 10 μg) inhibited tumor growth after tumor implantation. Significantly, the HER-2/neu DNA vaccine plus AC hmwPSs further reduced tumor sizes and further prolonged the survival of the mice compared to that of the HER-2/neu DNA vaccine alone (Fig. 8B). These data strongly illustrated that AC hmwPSs can enhance the therapeutic efficacy of HER-2/neu DNA vaccine against p185neu-expressing MBT-2 tumors *in vivo*.

### AC hmwPSs polarized the Th1 responses induced by HER-2/neu DNA vaccination

**Fig 9:**
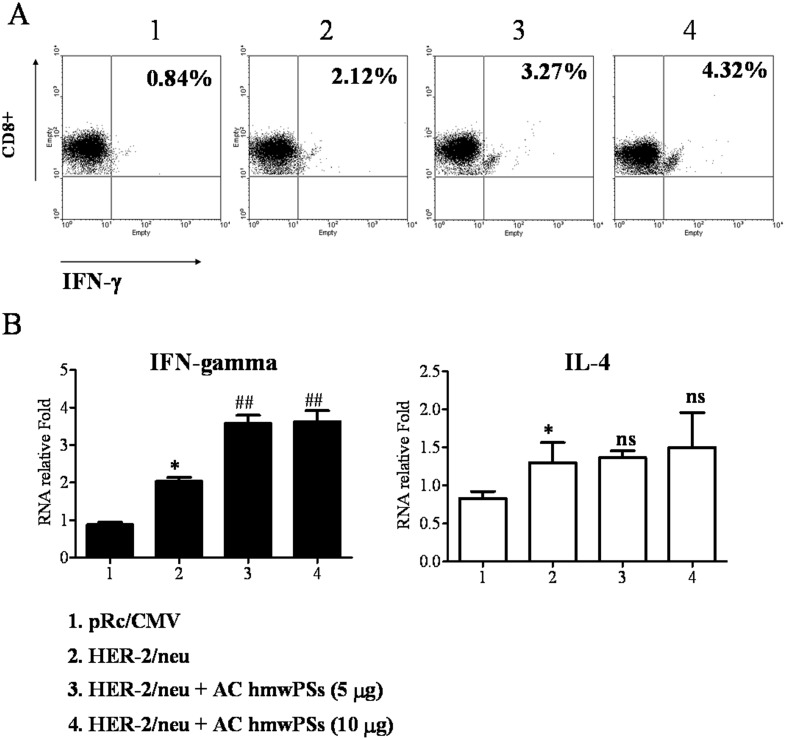
AC hmwPSs polarized the Th1 responses induced by HER-2/neu DNA vaccination. The tumor-bearing mice were treated with AC PSs, HER-2/neu DNA vaccine, or the combination as indicated for 6 days. Then, total inguinal LN cells were isolated from various vaccinated mice and incubated with recombinant HER-2/neu protein (10 μg/mL) for 16 h. (A) The production of IFN-γ by stimulated CD8^+^ T cells was detected by intracellular staining and flow cytometry. The percentage of CD8^+^IFN-γ^+^ cells in total CD8^+^ T cells is shown in each graph. (B) CD4^+^ T cells were purified from the total LN cells. The production of IFN-γ and IL-4 by stimulated CD4^+^ T cells were determined by quantitative real-time PCR. The data are normalized to HPRT expression in each sample and shown as the mean + SD of five mice. *p<0.05 (*Mann*–*Whitney U test*) are comparisons of treatment between HER-2/neu DNA vaccine and control. ^NS^ *p*>0.05; ^##^ *p*<0.05 are *comparisons of treatment between* HER-2/neu DNA *vaccine + AC hmwPSs and* HER-2/neu DNA *vaccine alone*. All of the data are representative of three independent experiments.

In Fig. 4, we found that DCs activated by AC hmwPSs increased T cell proliferation and preferentially skewed Th1 differentiation *in vitro*. As the Th1 response is necessary for tumor clearing, we then examined whether AC hmwPSs also augmented Th1 responses induced by the HER-2/neu DNA vaccination *in vivo*. Total lymph node cells isolated from mice with various treatments were incubated with recombinant HER-2/neu protein, and then IFN-γ production by CD8^+^ T cells was detected using intracellular staining. It was clear that more CD8^+^ T cells from mice immunized with HER-2/neu DNA vaccine plus AC hmwPSs secreted IFN-γ than did cells from mice immunized with the HER-2/neu DNA vaccine alone (Fig. 9A). Furthermore, we determined the expression of IFN-γ (Th1) and IL-4 (Th2) in CD4^+^ T cells by quantitative real-time PCR. Consistent with the results in CD8^+^ T cells, the vaccination with HER-2/neu DNA vaccine plus AC hmwPSs induced higher generation of IFN-γ in CD4^+^ T cells than was observed with either control vector, AC hmwPSs alone, or HER-2/neu DNA vaccine alone (Fig. 9B). In contrast, no IL-4 production could be detected in all tested cells. These data indicated that AC hmwPSs facilitate HER-2/neu-specific Th1 responses induced by HER-2/neu DNA vaccination and thereby enhance the anti-tumor efficacy of HER-2/neu DNA vaccine.

## Discussion

In this study, we characterized the activity of AC on the immune function of DCs. We discovered a promoting effect of AC PS on DC activation. Furthermore, we identified that a high-molecular weight fraction of AC PS majorly contributed to the induction of DC activation and function. Thus, for the first time we report here that AC PS has immunostimulatory activity on DCs and that AC hmwPSs may be a potent adjuvant.

AC is an endemic mushroom in Taiwan. Its crude extracts and pure compounds, such as polysaccharides, terpenoids, proteins, and phenol derivatives, possess a variety of health-promoting functions, including anti-hypertension, anti-inflammatory, antioxidative, anti-cancer, and immunomodulatory activities [23]. According to current literature, the majority of AC studies emphasize the inhibitory effect of AC on inflammation and tumor growth, especially the effect of small compounds isolated from AC [4]. Thus, our study about the immune-enhancing effect of AC PS uniquely promotes AC research. AC PS can now be added as a new immunostimulator for DCs.

The biological activities of AC PS have been revealed, including anti-hepatitis B virus [24], anti-tumor [25], anti-angiogenesis [8,26,27], protection from hepatic injury [28], and anti-inflammation [29–31]. In addition, AC PS has been shown to modulate the immune system for inhibiting infection by Schistosoma [32,33]. In these reports, the researchers use total AC PSs for their studies. Interestingly, Kuo *et al*. demonstrated that the immunomodulatory effect of AC mycelia is mainly attributed to the 10–20 kDa polysaccharides [34], which is much smaller than the active hmwPSs in our study. This difference may be due to the different types of immune cells that were tested; Kuo *et al*. used diluted peripheral blood with mainly polymorphonuclear neutrophils (PMN), whereas we used pure DCs. However, Yang *et al*. showed that only the AC PSs with high molecular weight (> 100 kDa) increase the IL-12 production by mononuclear cells and have anti-angiogenic effect [8]. Interestingly, the immunostimulatory activity of hmwPSs has also been reported in other natural sources [35–38], in agreement with our data.

Regarding the DC research in the AC field, we previously reported that a triterpenoid isolated from AC promotes DC activation and Th2 differentiation [18]. This is the first study to examine the effect of AC on DC. Although we previously showed that AC can enhance the anti-tumor efficacy of a DNA vaccine in a mouse tumor model, we only used crude extract and did not examine the effect of AC on pure DCs [21]. In another work, Liu *et al*. also examined the regulatory role of AC PSs on DCs, but they observed that AC PS treatment on LPS-activated DCs enhanced IL-10 production and suppressed naive CD4 T-cell proliferation in an allogenic mixed lymphocyte reaction [39]. It is possible that our AC hmwPSs may be different from their PSs.

Many PSs have potential to stimulate DCs, such as Fucoidan, a well-known group of certain fucose-containing sulfated PSs [40–42]. Fucoidan has a backbone built of (1→3)-linked α-L-fucopyranosyl or of alternating (1→3)- and (1→4)-linked α-L-fucopyranosyl residues, but also include sulfated galactofucans with backbones built of (1→6)-β-D-galacto- and/or (1→2)-β-D-mannopyranosyl units with fucose or fuco-oligosaccharide branching, and/or glucuronic acid, xylose or glucose substitutions [43]. When we compared the composition and structure of fucoidan to AC hmwPSs, these PSs were quite different because AC hmwPSs have a backbone built of partial fucosylated 1,6-α-D-mannogalactan composed of a nonadecasaccharide repeating unit [9]. In addition, AC hmwPSs have less fucose than fucoidan. Thus, the mechanisms for DC activation may be distinct between these PSs.

AC hmwPSs activated the NF-κB and MAPK pathways (Fig. 5), which may be the mechanism by which it enhances DC function. A number of reports have shown this unique ability of PS [5]. However, other mechanisms, such as PI3 kinase and PKC, have also been reported to be involved in immunomodulation by mushroom polysaccharides [44]. It will be interesting to further investigate whether these pathways are also related to the DC activation by AC hmwPSs. To initiate these signaling events, we identified that TLR2 and TLR4 were required for the stimulatory effect of AC hmwPSs, but TLR4 might play a more important role than TLR2 (Fig. 6). Consistent with our findings, several types of PS can also activate immune cells via TLR2 and TLR4 [45–47]. Thus, AC hmwPSs are likely a new ligand of TLRs.

In vaccine development, DNA vaccines have undergone a number of technological improvements and have become attractive and promising in the field, especially for therapeutic vaccination against intracellular pathogens and cancer [48]. PSs have been used as adjuvants in DNA vaccines against infectious diseases, such as HBV [49,50], *Trypanosoma cruzi* [51], *Chlamydophila abortus* [52], and HIV[53]. Notably, we reported here for the first time that AC hmwPSs significantly enhance the effect of the HER-2/neu DNA vaccine against p185neu-expressing MBT-2 tumors *in vivo* (Figs. 8 and 9), suggesting the application of AC hmwPS in a therapeutic vaccine against cancer. In addition, our data also imply the benefit of AC PS in immunotherapy for HER-2-expressing breast cancer [54]. It would be valuable to test the adjuvanticity of AC hmwPSs in other types of vaccine, such as DC vaccines for cancer therapy.

Another interesting observation was that we identified the stimulatory effect of AC hmwPSs on mouse BMDCs. BMDCs can home to the gut and model gut DCs *in vitro* [55,56]. Thus, it is possible that AC hmwPSs could activate DCs in the gut and thereby enhance immunity, suggesting that AC hmwPCs may also be applied as a mucosal adjuvant.

In conclusion, AC hmwPSs have immunostimulatory activity on DCs. Our results emphasize again that AC can serve as an important biofactory because it contains various bioactive components for health promotion and medical application. Moreover, we provide evidence that AC hmwPSs may have the potential to be developed into adjuvants in immunology and vaccine development. Recently, polysaccharides have been applied as polymer adjuvants in vaccination because of the biocompatibility, biodegradability, and non-toxic properties, making them attractive candidates for substituting conventional adjuvants [57]. This concept may expedite the clinical application of AC PS in human and then enhance the efficacy of vaccine against infectious diseases and cancer.

## Supporting Information

S1 Fig The stimulatory effect of AC hmwPSs on DCs is not caused by endotoxin contamination. DCs were treated with PBS (control), LPS (100 ng/mL), or AC hmwPSs (10 μg/mL) in the absence or presence of polymyxin B (PMB). Supernatants were collected after 24 h. The amounts of IL-6, and IL-12 were determined by ELISA. The data shown are the mean ± SD of three samples. ^NS^ *p*>0.05; *p<0.05 (*Mann*–*Whitney U test*) are comparisons between PBS-treated and PMB-treated DCs. All of the results are representative of three independent experiments.
(TIF)

S2 Fig Destroyed AC hmwPSs lost the stimulatory activity on DCs. DCs were treated with PBS (control), AC hmwPSs (10 μg/mL), or trifluoroacetic acid (TFA)-lysed AC hmwPSs (10 μg/mL) for 24 h. Supernatants were collected after 6 h. The amounts of TNF-α, IL-6, and IL-12 were determined by ELISA. The data shown are the mean + SD of three samples. *p<0.05 (*Mann*–*Whitney U test*) are comparisons between AC hmwPS-treated and PBS-treated or TFA-AC hmwPS-treated and AC hmwPS-treated DCs as indicated. All of the results are representative of three independent experiments.
(TIF)

S3 Fig AC lmwPSs (< 100 kDa) had no effect on DC maturation. DCs were treated with PBS (control) or various AC PS fractions as indicated (10 μg/mL for each fraction) for 16 h. The expressions of CD40 and CD86 (gray-filled area) were determined by immunostaining and flow cytometry. All of the data shown were gated on CD11c^+^ cells. The black line represents staining with an isotype-matched control antibody. The level of expression is indicated as MFI in each graph. All of the results are representative of three independent experiments.
(TIF)

S4 Fig AC hmwPSs had no effect on cancer elimination in mouse MTB-2 tumor model. The p185neu-expressing MTB-2 cells were inoculated in mice as described in the Materials and Methods. After ten days, the tumor-bearing mice were treated with PBS (control) or two doses of AC hmwPSs (5 or 10 μg/mouse). The data shown are the mean ± SD of six to seven mice per group. (A) Tumor growth curve in mice after treatment. The tumor volumes were calculated as described in Materials and Methods section at the indicated day. (B) The Kaplan-Meier survival curve of mice (N = 5) after treatment. All of the results are representative of three independent experiments.
(TIF)
