# Supplementary figures and images for: The Adjuvant Effects of High-Molecule-Weight Polysaccharides Purified from Antrodia cinnamomea on Dendritic Cell Function and DNA Vaccines
Source: PLoS One. 2015 Feb 27;10(2):e0116191. doi: 10.1371/journal.pone.0116191 (PMC4344241; doi:10.1371/journal.pone.0116191)

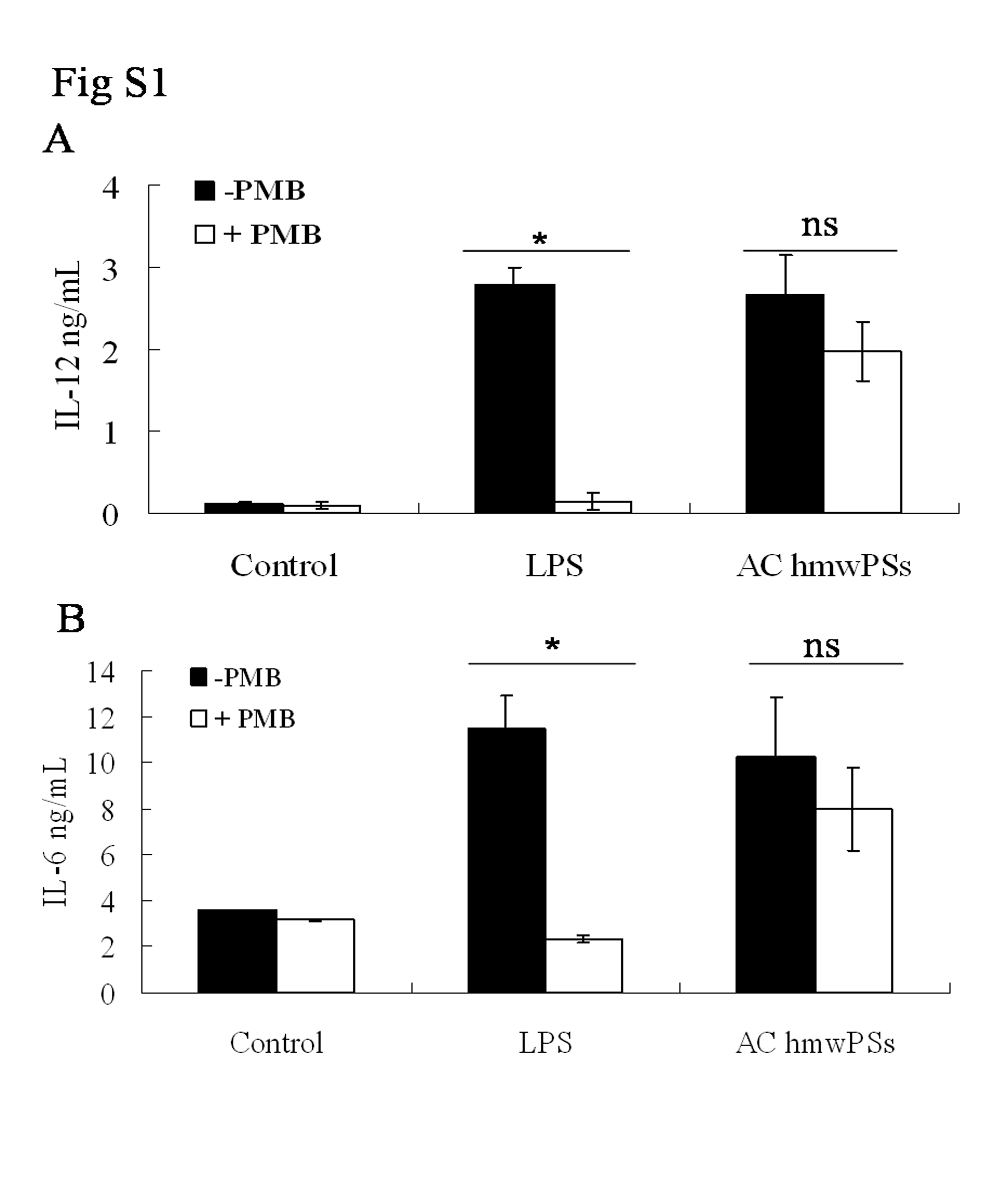

Supplement: S1 Fig — DCs were treated with PBS (control), LPS (100 ng/mL), or AC hmwPSs (10 μg/mL) in the absence or presence of polymyxin B (PMB). Supernatants were collected after 24 h. The amounts of IL-6, and IL-12 were determined by ELISA. The data shown are the mean ± SD of three samples. NS p>0.05; *p<0.05 (Mann–Whitney U test) are comparisons between PBS-treated and PMB-treated DCs. All of the results are representative of three independent experiments. (TIF) [file pone.0116191.s001.tif]

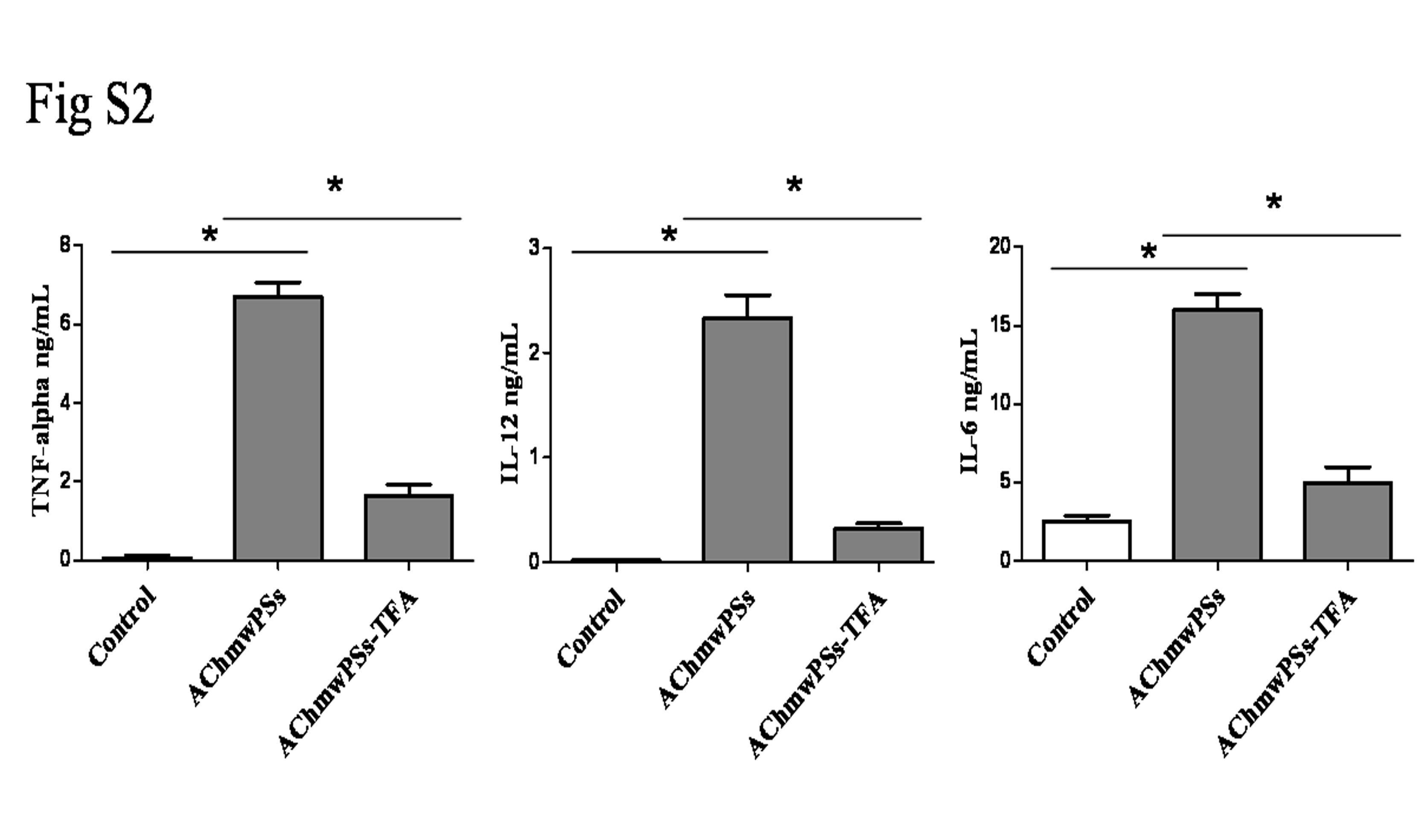

Supplement: S2 Fig — DCs were treated with PBS (control), AC hmwPSs (10 μg/mL), or trifluoroacetic acid (TFA)-lysed AC hmwPSs (10 μg/mL) for 24 h. Supernatants were collected after 6 h. The amounts of TNF-α, IL-6, and IL-12 were determined by ELISA. The data shown are the mean + SD of three samples. *p<0.05 (Mann–Whitney U test) are comparisons between AC hmwPS-treated and PBS-treated or TFA-AC hmwPS-treated and AC hmwPS-treated DCs as indicated. All of the results are representative of three independent experiments. (TIF) [file pone.0116191.s002.tif]

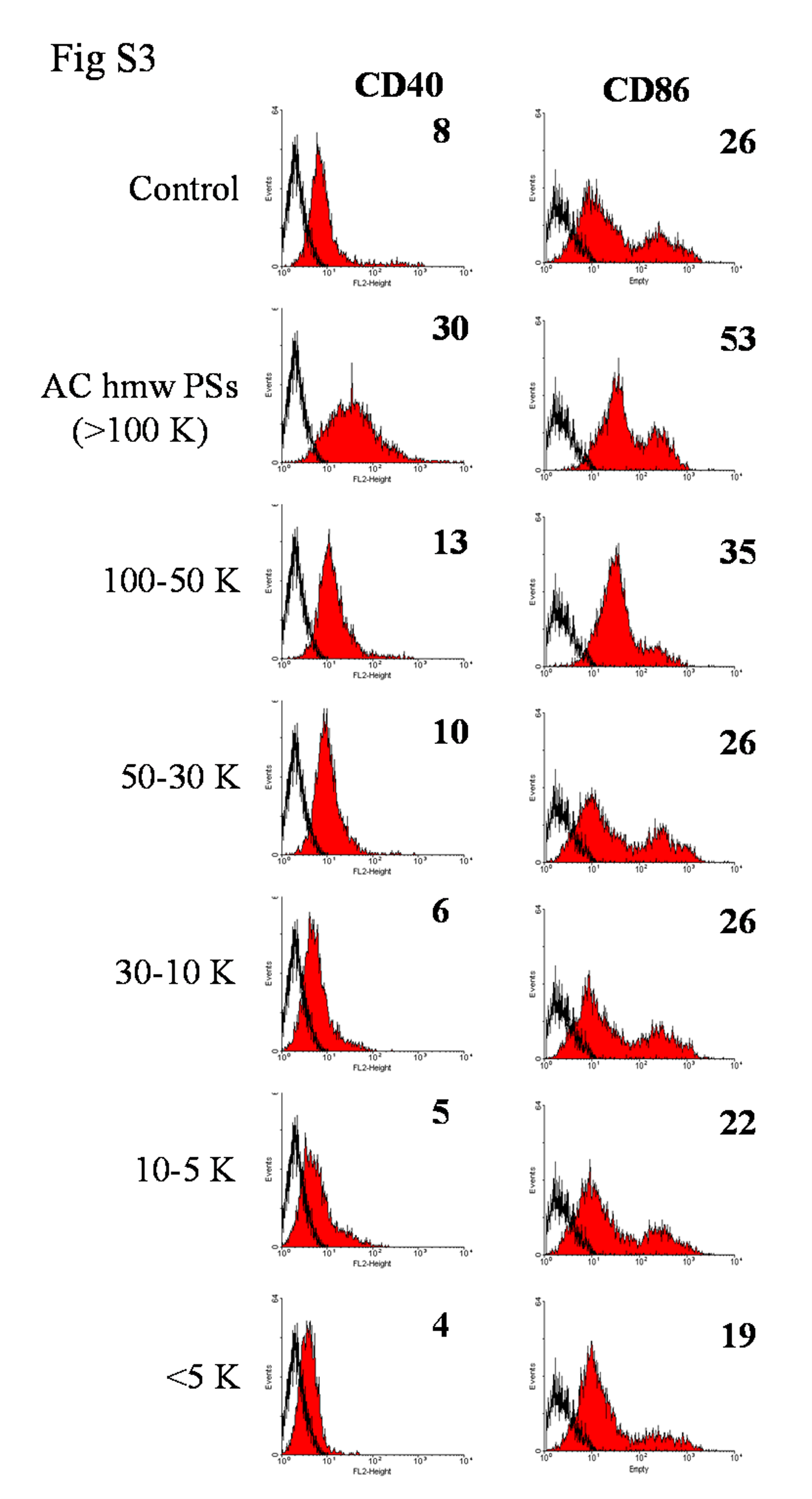

Supplement: S3 Fig — DCs were treated with PBS (control) or various AC PS fractions as indicated (10 μg/mL for each fraction) for 16 h. The expressions of CD40 and CD86 (gray-filled area) were determined by immunostaining and flow cytometry. All of the data shown were gated on CD11c+ cells. The black line represents staining with an isotype-matched control antibody. The level of expression is indicated as MFI in each graph. All of the results are representative of three independent experiments. (TIF) [file pone.0116191.s003.tif]

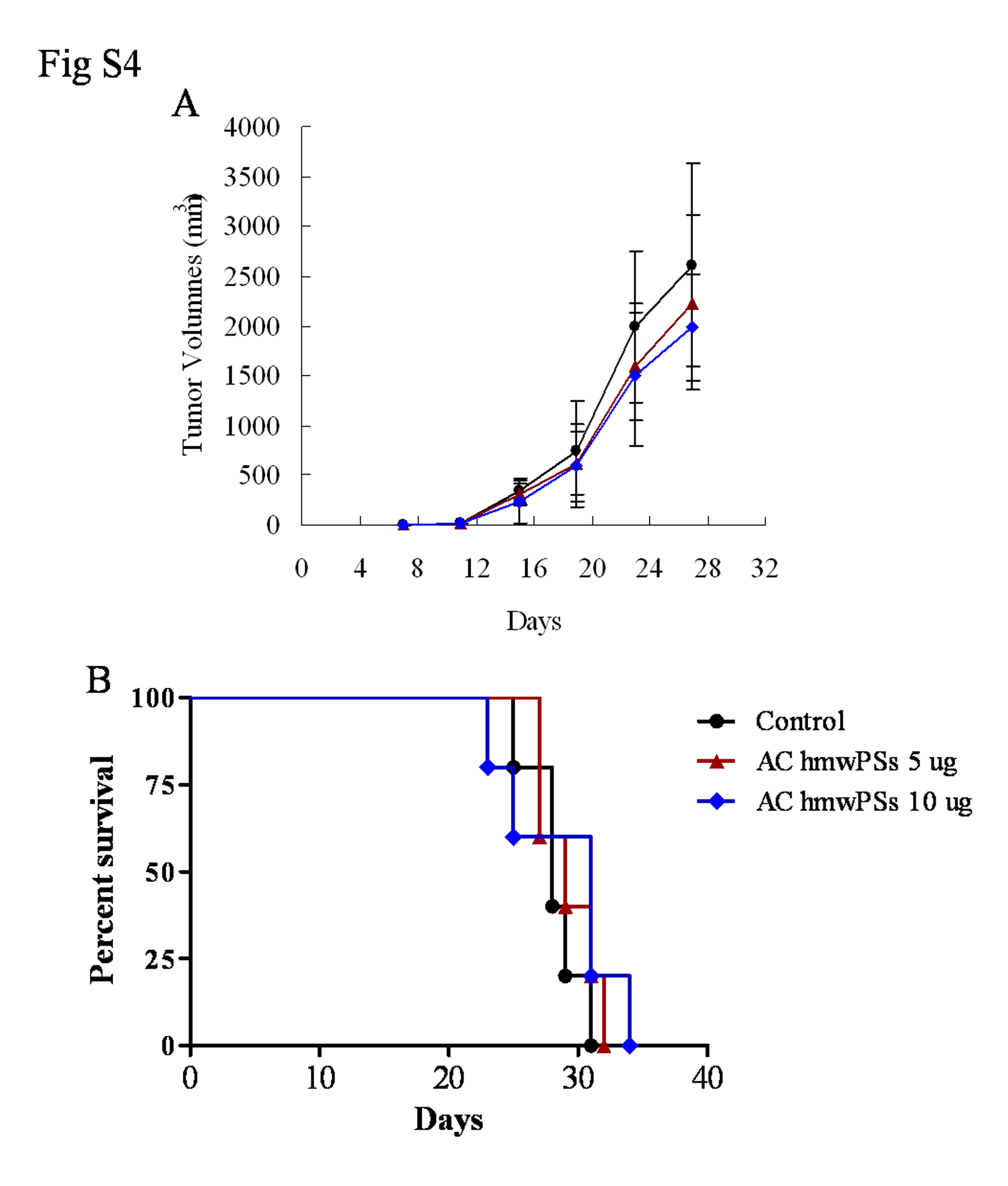

Supplement: S4 Fig — The p185neu-expressing MTB-2 cells were inoculated in mice as described in the Materials and Methods. After ten days, the tumor-bearing mice were treated with PBS (control) or two doses of AC hmwPSs (5 or 10 μg/mouse). The data shown are the mean ± SD of six to seven mice per group. (A) Tumor growth curve in mice after treatment. The tumor volumes were calculated as described in Materials and Methods section at the indicated day. (B) The Kaplan-Meier survival curve of mice (N = 5) after treatment. All of the results are representative of three independent experiments. (TIF) [file pone.0116191.s004.tif]
